# Educational Interventions to Improve Safety and Health Literacy Among Agricultural Workers: A Systematic Review

**DOI:** 10.3390/ijerph17031114

**Published:** 2020-02-10

**Authors:** Madalina Adina Coman, Andreea Marcu, Razvan Mircea Chereches, Jarkko Leppälä, Stephan Van Den Broucke

**Affiliations:** 1 Department of Public Health, College of Political, Administrative and Communication Sciences, Babes-Bolyai University, 400376 Cluj-Napoca, Romania; andreea.marcu@publichealth.ro (A.M.); razvan.m.chereches@gmail.com (R.M.C.); 2 Natural Resources Institute Finland (Luke), Production Systems, 00790 Otaniemi, Finland; jarkko.leppala@luke.fi; 3 Psychological Sciences Research Institute, Université catholique de Louvain, 1348 Ottignies-Louvain-la-Neuve, Belgium; stephan.vandenbroucke@uclouvain.be

**Keywords:** farm safety, agriculture, health literacy, education, safety, review

## Abstract

Health and safety education for farmers has the potential to increase the level of health, safety literacy, and thereby improve farmers’ health and quality of life. The aim of this paper is to provide a systematic review of the published literature documenting different educational interventions for agricultural workers that have the improvement of health and/or safety literacy as an outcome. A systematic search was conducted in PubMed, Embase, Scopus and PsycINFO databases for articles focusing on educational interventions for farmers’ health and safety. From the 3357 initial hits, 36 unduplicated records met the inclusion criteria. The articles included in the review used educational interventions for farmers with the purpose of preventing farm-induced diseases and injuries, increasing the health and well-being of farmers, and promoting good manufacturing practices. The educational approaches considered varied from lectures, videos, newsletters, games, and community fairs, to involving the community in designing the intervention and training farmers to deliver the intervention to the community. Interventions that used evidence-based theories, which took into account cultural aspects and individual factors, used biomarkers as a behavior change measurement, and involved the community in the development of the intervention had the best results in terms of behavior change. The strategies of educational interventions identified in this review that produced good results have the potential to inform future researchers and policy makers in the design and implementation of public health interventions, programs and policies to improve the health of farmers and their families.

## 1. Introduction

Research findings and work accident statistics in the agricultural field show that while almost half of the labor force at a global level works in agriculture, agricultural work is hazardous [1,2]. The hazards not only include occupational injuries, but also occupational diseases that might occur due to the nature of agricultural work and work environment. Therefore, initiatives to improve farm health and safety are important. Farm health and safety initiatives include a broad array of actions related to the 3Es of health and safety: Education, Engineering, and Enforcement [3,4]. All of these aim to increase farm safety and have an effect on improving the health of agricultural workers [1].

Out of the three strategies mentioned, education aims to increase farmers’ health and safety by encouraging them to adopt health-enhancing behaviors. To achieve this, education not only tries to enhance knowledge, but also skills, attitudes and practices that serve to prevent accidents and maintain good health. While the effects of health and safety education are often measured by distal outcomes such as injury and disease prevalence, a more direct outcome of health education is health literacy [5,6]. Health literacy is defined as the competence of individuals and communities to access, understand, appraise and apply information that is relevant to take decisions regarding one’s health [6]. A related notion is safety literacy, which can be viewed as a content-specific form of literacy in a health context that focuses on the safety of the patient/worker [5,7,8]. A number of studies have shown that low health and safety literacy is a problem not only among the general population, but also among underserved populations [9,10,11,12]. Lower levels of health literacy are linked with low levels of education and literacy, and overall poorer health outcomes [13]. As such, education has the potential of improving general literacy levels as well as health literacy and safety literacy, and ultimately of leading to a better health and quality of life [14].

Health literacy and safety literacy are not the sole responsibility of individuals, equal attention should be given to governments, agencies, professionals and policy makers who design interventions, programs and policies [15]. As an outcome of health education, health literacy is a pivotal concept for public health and health promotion [16,17] as it relates to the environmental, political and social factors that determine health [18] by including not only personal, but also societal and environmental benefits [6]. The growing recognition of its importance has led to policies to address low health literacy through different strategies, such as: measuring and monitoring the level of health literacy in populations, developing educational programs and interventions to enhance health literacy, and including health literacy in the training of health service providers to increase safety and safety literacy [19,20].

While the link between health education and health literacy is widely acknowledged, it is seldom addressed in research and practice with regard to farmers’ safety and health. Nevertheless, increasing the level of health and safety literacy through education has a great potential to improve the health, safety and quality of life of farmers and their families [21]. Among the farming population, there are specific types of workers, such as migrants, who are often employed in agriculture [22] and as such are exposed to occupational hazards and risks [23]. Low levels of education, diversity of language, young age and lack of experience are characteristics that differentiate the specific needs in terms of agricultural training and approaches to health safety training for this type of agricultural worker [24,25]. Apart from migrants, there are other populations that are affected by agriculture hazards, such as women and youth. They do not always come in direct contact with agricultural work, but are nonetheless affected by it [26]. The aim of this paper is to present a systematic review of the published literature on educational interventions for agricultural workers that have the improvement of health and/or safety literacy as an outcome. The objectives of the review are (1) to identify educational strategies to improve farmers’ health and/or safety literacy; and (2) to assess the extent to which educational interventions can be used to inform public health policies aimed at increasing farmers’ health and safety literacy.

## 2. Materials and Methods 

This systematic literature review followed the Preferred Reporting Items for Systematic review and Meta-Analysis Protocols (PRISMA-P) [27,28]. The Cochrane Handbook for Systematic Reviews of Diagnostic Test Accuracy was followed for the search criteria, data extraction, assessment of the methodological quality, analysis and interpretation of the results [29]. The protocol for this systematic literature review was registered on PROSPERO under the ID: CRD42018117843 since November 2018.

### 2.1. Search Strategy for the Identification of Studies

A systematic search was conducted in PubMed, Embase, Scopus and PsycINFO databases to look for articles focusing on educational interventions for farm safety in November and December 2018. These databases were chosen on account of the high number of indexed journals that cover multidisciplinary fields, both in agriculture and connected fields. Four main themes were specified for this literature review: health literacy, agriculture, farm safety and study design, and their associated search terms using Boolean operators (Table 1). Specific search strategies were developed for each database, and subsequently pilot-tested. They consisted of using a combination of keywords and controlled terms from Medical Subject Headings, Embase Subject Headings, and Thesaurus of Psychological Index Terms (refer to Supplementary Materials Appendix 1). In addition, reference lists of all selected articles were manually screened for eligible papers that were not identified through the other search procedures, and the “cited by” button on Google Scholar was used to trace additional eligible papers.

### 2.2. Eligibility Criteria

For this review, all studies that focused on and had an outcome related to farm safety in terms of injury prevention or health disorders due to the nature of the work were taken into account, irrespective of their methodological quality. Studies containing interventions that only focused on improving knowledge and that did not provide some competencies or skills for the agricultural workers were not taken into consideration. Only studies that had an intervention which aimed to offer both knowledge and competencies or skills were included in the review, since health literacy refers both to understanding and applying the health information in order to make appropriate health decisions [30]. Interventions that focused on mental health and mental health literacy were also excluded due to the nature of concepts studied and the difference in the type of interventions for physical health and mental health [31].

The review included studies that had agricultural workers as participants, as defined in the Division 01 from section A—Agriculture, forestry and fishing from International Standard Industry Classification (ISIC) divisions A 011 Growing of non-perennial crops; A 012 Growing of perennial crops; A 013 Plant propagation; A 014 Animal production; and A 015 Mixed farming [32]. Based on the same classification, participants that fall under the categories A016 Support activities to agriculture and post-harvest crop activities; A 017 hunting and trapping; A 02 forestry and logging; A03 fishing and aquaculture; and nonagricultural industries were excluded, due to the differences in the nature of farming work [1].

Study designs selected for the review included, but were not limited to: randomized controlled trials (RCTs), laboratory and field experiments, quasi-experiments, and pre-and/or post-intervention designs. Descriptive studies and literature reviews of farm safety were excluded. Studies that had the abstract in English but the main body text in a different language and studies for which we could not retrieve the full text were also excluded.

No setting restriction was applied. Studies from all countries were taken into consideration if they met the eligibility criteria, as long as the articles had been published in English. No time limit was set as an eligibility criterion for the systematic literature review.

### 2.3. Study Selection and Data Extraction

Documents obtained via the initial search were downloaded and imported in CADIMA, an evidence synthesis tool and database for systematic reviews [33], which automatically identified and removed duplicates. The selection of papers was done by two independent reviewers. The interrater reliability (κ = 0.759) as calculated by CADIMA, suggesting substantial agreement between both reviewers [34]. Any disagreement between the reviewers was resolved through discussion.

The following data was extracted for each included article: (1) general details (title, authors, year of publication, country, and topic); (2) specific details (aim, design, sample size, type of farming, duration of intervention, types of statistical analysis performed); (3) intervention details (type of intervention, evidence-based intervention, concept of health literacy used, evaluation of intervention); and (4) outcomes (outcomes targeted, main results, main conclusions, limitations, future recommendations and policy implications).

The quality of studies included in the review was assessed by means of an adapted methodological quality rating system developed by Seymour and coworkers [35]. The rating system consisted of a score from 1–9 given to each article based on the strength of the design and methodology of the study (setting of the study, design, duration of intervention, times of exposure, sample size, random allocation to groups, statistical analysis, generalizability, and use of biomarkers). More details about the quality rating system is provided in Table 2. It was decided to use this system rather than others because farming is highly dependent on contextual factors and the research undertaken about this topic makes it difficult to avoid all biases.

## 3. Results

**Figure 1:**
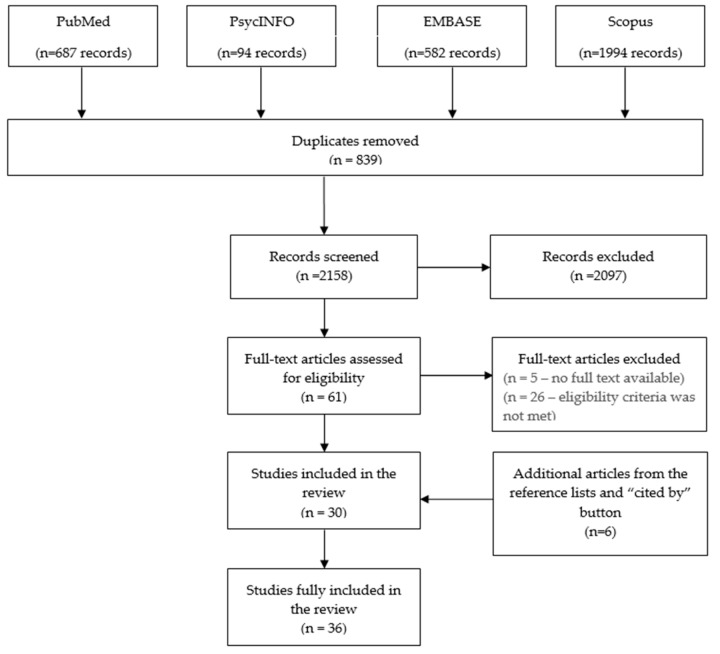
Review flowchart.

From the 3357 initial hits, 30 unduplicated records met the inclusion criteria. After searching the reference lists and “cited by” button on Google Scholar of these articles, six more studies were retrieved, giving a total of 36 articles to be included in the systematic review (Figure 1).

Of these 36 studies, a number of 12 studies focused on farm-induced diseases such as prevention of zoonosis [36,37,38,39,40], asthma [41,42,43], skin cancer [44], hearing loss [45], and good manufacturing practices for prevention of antimicrobial resistance [46] and microbiological contamination [47]. A number of two studies focused on agricultural health promotion [48,49] and 23 studies focused on accident and injury prevention concerning pesticide safety [50,51,52,53,54,55,56,57,58,59,60,61,62,63,64] and injury prevention among farmers [65,66,67,68,69,70,71]. The years of publication varied from 1996 [44] to 2018 [63]. Eighteen articles presented research performed in North America (16 in USA) [42,43,44,45,51,53,54,59,61,64,65,66,67,68,69,70], one in Canada [38], and one in Nicaragua [60]); eight articles presented research performed in Asia (three in Thailand [62,63,71], two in India [52,57], one in Laos [37], one in Cambodia [39], and one in Vietnam [40]); four articles presented research from South America (two from Ecuador [55,58], one from Bolivia [56], and one from Brazil [47]); three articles presented research from Africa (two from Tanzania [36,46] and one from Egypt [50]); two articles were from Australia [48,49], and one from Europe [41]. Of the studies considered for the review, six had used an RCT as a study design [36,39,61,63,64,69], 20 used a pre-post with control group [37,38,40,42,43,45,47,51,53,56,59,60,62,65,66,67,68,70,71], and 10 used a pre-post design without control group [44,46,48,49,50,52,54,55,57,58].

The duration of intervention in the selected studies ranged from one hour to five years. Some studies’ intervention consisted of a single exposure, whereas others had repeated exposures. A full overview and description of the articles included in the review is available in Supplementary Materials Appendix 2.

### 3.1. Study Quality

Using the adapted methodological quality rating system developed by Seymour [35], each article received a score based on the strength of the design and methodology. As shown in Table 2, a score below 5 indicated that the quality of the study was weak, a score between 5–6 that it was moderate, a score between 7–8 that it was strong, and a score of 9 meant that the study had a very strong quality. 

From the studies included in this review, four had a weak methodological quality [47,53,57,65], 19 had a moderate methodological quality [38,40,41,42,44,45,46,50,51,52,54,55,56,58,61,63,64,69,71], and 13 had a strong methodological quality [36,39,43,48,49,59,60,62,66,67,68,70]. None of the studies received a score indicating a very strong methodological quality, since none of them produced results that were generalizable. The strength of the methodological quality was not better in any specific country or in studies dealing with a specific topic. Overall, the results showed great variability with regard to the methodological quality of the studies across contexts and types of interventions. 

### 3.2. Studies Focusing on the Prevention of Farm-Induced Diseases

Studies focusing on the prevention of farm-induced diseases included interventions on the prevention of zoonosis (a type of disease that is transmitted to humans from animals), asthma, skin cancer, and hearing loss, and good manufacturing practices for dairy products. 

Of the five studies focusing on prevention of zoonosis, three had been performed in Asia [37,39,40], one in Africa [36] and one in Canada [38]. Two of the studies involved farms with large ruminant animals as livestock [37,38], two looked at poultry farms [39,40] and one at pig farms [36]. The number of participants in the interventions varied from 176 [38] to 827 [36] and participants in all the studies were farmers that worked directly with the animals. Two of the studies were classified as RCTs [36,39], the other three used a pre-post design with a control group. Of the latter, one applied random sampling of the groups [37], while both others used a convenience sample [38,40].

The duration of the interventions ranged from two days [40] to three years [37]. For two of the studies, the intervention consisted of a single exposure [36,40], while the remainder used repeated exposures [37,38,39]. The methods and instruments used for zoonosis prevention included training sessions using videos, leaflets, booklets, posters, and training manuals [36,40], as well as farm visits and collaboration [37], learning games [38], and training peers to deliver the intervention [39]. One of the studies mentioned the Theory of Planned Behavior as a basis for the intervention [40], while three of the studies had used community-based participatory research approaches (although this was not specifically mentioned in the articles [37,39,40]). One study did not mention any evidence-based theory to support the intervention [36]. None of the studies focusing on zoonosis mentioned the health literacy concept, but one study tailored the intervention to both literate and illiterate people [36].

The findings of these studies showed that peer-learning approaches and using villagers in teams of trainers were successful in preventing zoonosis [37,38,39,40]. In contrast, education consisting of just lectures, videos or training manuals had weaker results in terms of behavior change [36]. The recommendations deriving from these studies include the use of participatory approaches involving local healthcare workers and administrators to ensure acceptance and sustainability of zoonosis prevention programs [37,39,40], and the use of longitudinal studies to evaluate the effects over time. Neither of the studies mentioned a policy component, although three studies mentioned involving different institutions from the community in developing prevention programs [37,39,40].

Of the studies focusing on asthma, two [42,43] had been performed in North America and one in Europe (Germany) [41]. All three the studies focusing on asthma and respiratory disease had a pre-post intervention with control group design. Their samples ranged from 68 [42] to 308 participants [43], all of whom were farmers. Only one study had the participants randomly selected and blinded [43]. For two of the studies, the duration of the interventions was one day with a single exposure [41,42], for the third study it was five years with repeated exposure [43]. The methods and instruments used for the interventions included presentations on work-related asthma and protective equipment [41], presentations on work-related asthma and agricultural causes, spirometry testing, respirator demonstrations and fit testing, exposure reduction strategies, and barriers to personal protective equipment use [42]. The third study had an intervention with multiple components including clinical screening services, educational support, farm audit, and incentives [43]. Only one study that targeted respiratory diseases used an evidence-based theory to develop the intervention [43], and none of the studies mentioned the concept of health literacy in their interventions. The findings of the interventions on respiratory conditions show that providing information on work-related hazards and demonstrating proper use of protective equipment can lead to short-term improvements in knowledge and self-reported behaviors [42], but that long-term behavior changes require a multidimensional approach [43]. None of the studies mentioned a political component to improve the respiratory work-related hazards.

The article focusing on skin cancer [44] was performed in North America and presented a pre-post study without a control group involving 1310 farmers randomly selected on the basis of eligibility criteria. The duration of intervention was not specified, it was only mentioned that it took place repeatedly. The article did not refer to an evidence-based theory as a basis for the intervention, which consisted of multiple components including television, radio, and newspaper announcements as well as setting up information and screening booths at county fairs manned by family physicians, nurses and American Cancer Society staff. The findings of the study suggest that community-based educational interventions can build on farm families’ established routines of personal preventive behaviors towards skin cancer. The health literacy concept and political component were not mentioned in this study.

The study focusing on hearing loss [45] was performed in North America had a pre-post study design with a control group (convenience sample) involving 25 farmers. The duration of the intervention was not specified, it was only mentioned that it took place two times. The intervention was based on the Theory of Planned Behavior and used videos, farm assessment and personalized information for noise exposure reduction on farms. The intervention was successful in increasing the use of hearing protection, and led to the recommendation to use theory-based interventions and strategies that support partnerships between farmers and the community. The health literacy concept was not mentioned, but there was a political component in the sense that multidisciplinary collaborations for preventing hearing loss in farming settings was recommended.

The two studies evaluating good manufacturing practices focused on prevention of antimicrobial resistance and prevention of microbiological contamination for farmers working in the dairy industry. One article was conducted in Africa [56] and one in South America [57]. The study focusing on antimicrobial resistance [56], had a pre-post design without a control group, and involved a convenience sampling of 162 participants (men and women). The duration of the intervention was not specified and a single exposure to the intervention was mentioned. The intervention, which was not based on any specific evidence-based theory, consisted of education about the risks of contaminated milk and on practices on how to properly pasteurize milk and antibiotic dosage using thermometers and measuring tapes. The study showed that the intervention led to some improvement in the use of these materials (women used the thermometers more), but not in the knowledge about the importance of using them. A lack of education was found to significantly influence the use of materials and understanding of the concepts. Although the intervention itself did not take health literacy into account, the concept of health literacy was mentioned in the recommendation section, in the sense that materials should be tailored to the farmers’ literacy level and cultural beliefs. Localized, cultural and community-specific interventions were recommended to increase the farmers’ health literacy levels. The study did not mention any policy implication. The article focusing on improving good manufacturing practices to avoid microbiological contamination [47] was conducted in South America. It had a pre-post design with a control group study without mentioning the sampling method or the number of participants. The duration of the intervention was 1 hour and it consisted of a single exposure to the intervention. The methods and instruments used were not based on any specific evidence-based theory and the intervention consisted of a course using illustrative cards on easy-to-apply solutions on the improvement of the milking procedure with practical illustration. The study showed that although some improvements were observed, the intervention had limited results, since its principles were not incorporated in the work routine and in the attitudes of the participants. The study did not mention health literacy or any policy implication.

### 3.3. Studies Focusing on Agricultural Health Promotion

The two articles that focused on the general health and well-being of farmers discussed improving nutrition for a better health [48,49]. Both studies had been conducted in Australia, used a pre-post design without a control group, and involved a convenience sample, one consisting of 99 participants [48] and the other of 321 participants [49], both using families as agents of change. Both interventions lasted for three years and involved repeated exposure (meetings once per year). The methods and instruments used for the interventions consisted of workshops on agricultural health and safety topics, newsletters, a supermarket tour, health assessments by means of an individual manual for each participant [48], and workshops on health issues that are prevalent in farming and among rural populations, using written and visual materials and physical assessments [49]. Both interventions were based on the Theory of Planned Behavior and Kolb’s experiential learning model. Both studies demonstrated a significant improvement in health indicators, suggesting that education, evidence-based information, shared learning, and reinforcement of positive health behaviors lead to behavioral change and a better health. One of the studies urged for intersectoral collaboration and ownership as a political component and recommended increasing health literacy through education for improved health and knowledge outcomes for farm families [49].

### 3.4. Studies Focusing on Accident and Injury Prevention 

Studies focusing on accident and injury prevention tackled poisonings due to pesticide use and injuries related to farm work (eye injuries and musculoskeletal injuries).

Pesticides are substances that are used for pest and weed control in agriculture and that have toxic effects for humans if they are not handled correctly [4]. Of the 15 studies targeting pesticide safety, most had been performed in North America [51,53,54,59,60,61,64], followed by Asia [52,57,62,63], South America [55,56,58] and Africa [50]. The crops that were involved were strawberries [64], rice [62], maize [60], dairy products [61], alfalfa and Indian white corn [59], and cotton [57], whereas for nine studies the type of crop was not mentioned in the publication [50,51,52,53,54,55,56,58,63]. The number of participants involved in the studies varied between 50 [57] and 1200 [60]. Most of the studies focused on pesticide safety delivered directly to farmers. One study focused on women only [64]; while four studies included both parents in the intervention [50,53,54,64]. Of the latter, two focused on migrant families [53,54]. With regard to the study design, three studies used an RCT [61,63,64], six studies used a pre-post comparison without a control group [50,52,54,55,57,58], and six studies used a pre-post comparison with a control group [51,53,56,59,60,62]. From the pre-post study designs, one study applied a random selection of participants and random assignment to intervention and control groups [62], while two studies randomly selected the villages for the intervention [50,52], and the other ones used convenience samples.

In terms of duration, the interventions for pesticide safety ranged from a three hour session [61] to a five-year intervention [59], with two of the studies not mentioning the duration of the intervention [52,58]. Most of the interventions used repeated exposures ranging between weekly to monthly sessions [50,51,54,55,56,57,58,59,60,62], yet in three studies the intervention involved a single exposure [52,53,61], and two studies did not mention if the intervention was offered once or repeatedly [63,64]. The methods and instruments used for the interventions varied in theoretical and practical education sessions on pesticide handling and storage [50,51,52,53,54,56,57,61,63], some of which involved promoters in the community [51,54], to community-based participatory (CBPR) activities such as puppet shows, socio-dramas, games, interactive exercises, drawings, forums, mural painting, relaxation exercises and body awareness sessions [55]. Community fairs, organizing a health week, group discussions, farm visits [58,62], train-the-farmers to further train their neighbors on pesticide safety [59,60], and modifying the environment by providing warm water, soap, and protective clothing [64] were also mentioned. In terms of the theoretical basis for the intervention, 11 articles mentioned behavioral theories such as the Theory of Reasoned Action [51], the Theory of Planned Behavior [61], the Diffusion of Knowledge Theory [56,59], the Trans-Theoretical Model [53], the Health Belief Model [54,62,63], the conceptual model for Community-Based Participatory Research [55,59,63,64], or the conceptual framework developed by WHO [58]. Four articles [22,24,29,31] did not mention any evidence-based theory for the development of the interventions. The concept of health literacy was not mentioned in any of the studies, but (general) literacy was mentioned in seven of them [50,51,52,53,54,56,57], two of which tied literacy with health education and reducing poisonings from pesticide handling [56,57].

The reported outcomes of the interventions varied widely as a function of the type and duration of the intervention, the exposure to the intervention, the use of a comparison group for the study, and the method of evaluation used. The strongest results in terms of behavior change towards pesticide safety were reported for studies that used community participatory methods [59,64], based their intervention on an evidence-based theory [53,54], used a control group to evaluate effects [56,61,62], and considered not only self-reported indicators such as knowledge and skills but also biomarkers as outcome indicators [59,60,63]. Education in the form of lectures or videos without involving the community proved to have limited results, mostly on increasing knowledge, but not on behaviors [50,51,61]. The recommendations for the improvement of pesticide safety that were derived from the studies are to offer continuous safety education from an early age that is culturally appropriate, especially for illiterate people [50,51,52,57], to use evidence-based research and community participatory approaches for the design of the intervention [51,55,56,58], and to measure outcomes by means of biomarkers in addition to observation of real behavior change [62]. Of the 15 studies, five also referred to a political component [51,55,56,57,62], mentioning the importance of working with local authorities and community farmers to translate the results of the interventions into appropriate policy changes to improve pesticide safety by offering support through education and policy regulations.

From the seven articles dealing with the prevention of eye and musculoskeletal injuries among farmers, six presented research performed in North America [65,66,67,68,69,70] and one presented study conducted in Asia [71]. Four of the studies focused on injuries from fruit harvesting on migrant populations (eye injuries) [65,67,68,70], and three on injuries in animal and crop farming (machine injuries) [66,69,71]. The number of participants in the studies varied from 108 [68] to 786 [70]. One study focused on using the fathers as change agents for children’s behavior [69], while all other studies focused directly on farm workers. Six studies used a pre-post design with a control group, three of which used random allocation to intervention and control groups [66,70,71] and three used a convenience sample [65,67,68]. One study used an RCT as a study design [69].

The duration of the interventions for injury prevention ranged from one day [65] to three years [66], while two of the studies did not mention the duration of the intervention [69,70]. Only one of the studies used a single exposure to the intervention [65], the other six mentioned repeated exposures. The methods and instruments used for the interventions included video presentations (fotonovelas), live demonstrations of lifting techniques, the distribution of educational pamphlets [65], live discussions with health professionals, and farm visits [66,71]. Some interventions relied on trained peers to deliver the information to the entire working crew [67,68,70] or on members of the family to pass on information to other family members [69]. Two studies did not mention an evidence-based theory as a basis for developing the intervention [65,66]; the other ones used the Theory of Planned Behavior [69] and community-based models such as the Community Health Worker Model [67,68,70] and the Community-based Participatory Research conceptual model [71]. The concept of health literacy was explicitly mentioned in one study [65], which connected the concept with education for health improvement. Two other studies [67,68] designed the intervention for people with low levels of literacy and education. The remaining four studies on this topic did not mention health literacy or general literacy in their reporting.

The findings of these studies show that using a community-based participatory approach and involving peer educators [67,68,70,71] or family members [69] as trainers have good results in changing preventive behavior. On the other hand, using just information that is not adapted to the population [65] does not produce real changes, and using a self-reported measure of injury does not produce conclusive results [66]. The recommendations for the improvement of injury prevention deriving from these studies is that community-based participatory methods, such as peer- or family-led education, involving peers as role models for developing and delivering culturally appropriate, occupation-specific interventions [68,69] is recommended, as is using third party data instead of self-reported data to measure effects [45]. The policy component was mentioned in only one study [70], recommending policy changes to reduce eye injuries.

## 4. Discussion

The aim of this systematic literature review was to assess different educational interventions for agricultural workers that have the improvement of health and/or safety literacy as an outcome.

The quality assessment of the studies testing interventions focusing on improving health and/or safety literacy showed that although the majority of studies had a moderate to good methodological quality, none of the studies could be considered as having a very strong study design, mainly because none of them had results that could be generalized in the field. However, generalizability is hard to achieve in this type of research and setting, as farms, farmers, types of crops and farming techniques are so diverse in different parts of the world, and as it is nearly impossible to randomize contextual factors such as weather or market prices in agriculture [57]. All these factors can influence how an intervention is implemented and how participants will act, especially for longitudinal studies, which are the type of study most recommended in this field [48,59,68].

Our findings show that results of the interventions vary as a function of several factors. Some of these are contextual and cannot be controlled, but others are related to the type of intervention and design of the studies, and can be improved. The strongest results, irrespective of the type of farming undertaken or the behavior targeted by the intervention, were found for interventions that involve multiple exposures. Participants that are exposed more than once to different components of an intervention have better results in terms of behavior change [54,60,63].

Another finding that came out of this review is that providing education by methods that primarily aim at enhancing knowledge without involving the participants in the intervention have limited results. They mostly lead to an increase of knowledge, but not to a change of behaviors [50,51,61,65]. Moreover, cultural and educational aspects need to be taken into consideration when developing a farm safety and health intervention. This is especially the case for migrant populations and underserved populations with low levels of literacy, who have different needs in terms of occupational health training and health literacy [12]. The latter influences their ability to obtain, process and use information for their health. As such, improving health literacy leads to better health decisions, reduces risk factors, increases safety behaviors, and decreases the cost associated with care. Even if the concept of health literacy is not mentioned explicitly in many of the studies included in this review, the elements of the concept are taken into account and the recommendations deriving from the studies lean towards increasing health literacy levels among farmers as a way to increase farm safety [46,49,50,51,52,53,54,56,57,60,64,65,67,68].

Findings of studies reported in the literature have demonstrated that education has the potential to increase health literacy and farm safety [11], and that the results of health and safety education depend on how the concept of education outcome is defined and measured. While the literature shows that notable differences exists for task-based outcomes versus skilled-based outcomes, some interventions tended to focus more on selective domains from the education sphere [5]. Examples from the studies included in this review show that for some of the studies the education outcomes were simply self-reported measures of behaviors [57], whereas for others, several self-reported behaviors together with biomarkers were taken into account [59,60]. Our findings confirm that educational interventions that are based on evidence-based theories do not only produce better results, but also better lend themselves to the evaluation and measurement of effects, as they more clearly define the educational concepts involved and targeted [38,45,48,53,54,56,62,63,69]. Furthermore, apart from using evidence-based theories to develop interventions, using community-based participatory approaches have better results in terms of behavior change [37,39,40,43,55,59,62,64,67,68,70,71]. This can be explained by the fact that CBPR puts an emphasis on working with the community as full and equal partners in all phases of the intervention process. Involving farmers in the development of the intervention and training them to further deliver it to the community results in better adherence to the intervention, a stronger identification with the values promoted by the intervention and, ultimately, more chance of changing the behavior targeted by the intervention [72]. Moreover, using CBPR for the design of the intervention automatically takes into account cultural factors, contextual aspects and individual characteristics such as education and literacy, which is important to develop tailored interventions [59,68]. 

Another finding of this systematic literature review is that better evaluation results are found for studies with a longitudinal study design involving randomized control groups [36,39,69]. This finding is consisted with recommendations from the literature that encourage the use of randomized control groups over longer periods of time [73,74]. In addition, it was found that, if possible, biomarkers or third party data should be used complementary to self-reported data for achieving more accurate evaluation results. Studies that had a biomarker component showed more quantifiable and reliable responses and could as such be recommended as a golden standard when measuring results [36,37,42,43,45,46,47,48,49,59,60,63,66,67,68,70].

Finally, the findings of this systematic literature review also showed that interventions that used multidisciplinary or holistic teams, that involved intersectoral collaboration, and that were actively rooted in the local community led to more tangible results [37,39,40,45,49,51,55,56,57,62,70]. Such actions have the potential to inform public health policies focusing on the education of agricultural workers by involving local authorities, agencies and community farmers and to translate the results of interventions into appropriate policy changes.

The results of this review have some limitations. Although use of different electronic databases was made to find relevant studies, it is possible that not all relevant articles were included. Specifically, the exclusion of articles published in non-English languages may have resulted in some selection bias. In addition, for some of the articles, the results might not be reliable due to their poor research design and methodological quality, although most of the studies included in the review had moderate to strong quality and used adequate methods and criteria to evaluate the interventions. Due to the heterogeneity of the methodology of interventions and the difference in context, even among the same outcome topic, the results of the studies included in this review are not generalizable and do not guarantee the success of the intervention if transferred from one context to another. Therefore, this systematic literature review should be viewed as an overview of current research in the field and as a starting point for future research in the field of improving the health of farmers through interventions aimed to improve health and safety literacy.

## 5. Conclusions

The articles included in this review were concerned with educational interventions for farmers aimed at preventing farm induced diseases and injuries, increasing the health and well-being of farmers, and promoting good manufacturing practices. The educational approaches that were considered ranged from lectures, videos, newsletters, games, and community fairs, to involving the community in designing the intervention and training farmers to further deliver the intervention in the community.

Despite the limitations discussed, this review highlights some successful strategies and their potential to inform public health policies to improve health literacy and develop a safety culture among farmers. The findings show that interventions with the strongest results used the following strategies and components, regardless of the type of farming and behavior they targeted: they (i) used evidence-based theories as a basis to develop the intervention; (ii) involved the community in developing the intervention; (iii) took cultural aspects and individual factors into account, especially for migrant populations and families; and (iv) involved more than one moment of exposure to the intervention. To adequately test their effects, studies should ideally make use of a longitudinal design with a control group, and use biomarkers as well as observations in addition to self-report to measure behavior change. These findings offer valuable information since, to our knowledge, there is no synthetized evidence of the existent programs and strategies to improve health and safety literacy among farmers published so far. Using the results from our research, future practitioners can develop programs aiming to help farmers improve not only their knowledge, but also their competencies and skills in safety practices in agriculture. This aspect is important because it has the potential to help not just the farmers, but also their families, farming communities, and farm employees such as migrants to have better practices in agriculture and a better quality of life. The strategies and components of educational interventions identified in this review have the potential to inform future researchers and policy makers in the design and implementation of public health interventions, programs and policies to improve the health and well-being of farmers.

## Figures and Tables

**Table 1:** List of search themes and terms used for the searching strategies.

Search Themes	Search Terms
Health literacy	health literacy, education, literacy, safety literacy, competencies, competency, skill, skills, ability, abilities, capacities, capacity
Agriculture	agriculture, farm, farms, farmer, farming, farm worker, farmworker, ranch, dairy, dairies, dairying, greenhouse, orchard, livestock, livestock, farm animal, crop production, harvest, horticulture, agronomy, animal confinement, animal house
Farm safety	safety, safety practices, safety culture, safety measures, safety behaviors, health, health improvement, health promotion, health outcome, health condition, well-being, prevention, quality of life
Study design	allocated, assigned, clinical trial, comparative studies, controlled, controlled clinical trial, cross-over studies, experiment, intervention, placebo, pre-post, randomization, randomized controlled trial, RCT, observational, cohort, case-control

**Table 2:** Quality assessment (QA) of studies included in the review.

Rating	Definition	Study Description	Design and Methods
*	Weak	Many details missing (three or more of the following: setting, intervention design, duration, intensity, population, or statistical analysis), irrelevant design or methods.	Methodologic flaws (in statistical methods used, design of intervention, etc.).
**	Moderate	Some details missing (one or two).	Small sample size (<50) or short duration (<1 month).
***	Strong	Some details missing (one or two).	Larger sample size (≥50) and longer duration (>1 month).
****	Very strong	Very clear, all details provided.	Larger sample size (≥50), longer duration (>1 month), and all of the following criteria: population randomly allocated or matched for intervention or control, generalizable results, or validated assessment.

## Supplementary Materials

The following are available online at https://www.mdpi.com/1660-4601/17/3/1114/s1, Appendix 1―Search strategy, Appendix 2―List of articles included in the review.
